# The relationship between women climate change anxiety and sexual quality of life

**DOI:** 10.1097/MD.0000000000046973

**Published:** 2026-04-10

**Authors:** Hamide Aygör, Necibe Şeyda Tunali, Yusuf Bayar

**Affiliations:** a Faculty of Nursing, Necmettin Erbakan University, Konya, Turkey; b Health Services Vocational School, KTO Karatay University, Konya, Turkey; c Department of Medical Services and Techniques, Operating Room Services Program, KTO Karatay University, Konya, Turkey; d Faculty of Social Sciences and Humanities, Necmettin Erbakan University, Konya, Turkey.

**Keywords:** anxiety, climate, climate change, sexual quality of life, women

## Abstract

This correlational descriptive study was performed to investigate the relationship between the climate change anxiety levels and sexual quality of life of women. The study included 952 women. A personal information form, the Women Climate Change Anxiety Scale, and the Sexual Quality of Life – Female Questionnaire were used to collect data. The participants had a mean Women Climate Change Anxiety Scale score of 65.40 ± 11.51 and a mean Sexual Quality of Life – Female Questionnaire score of 34.25 ± 14.68. Age, marriage duration, education status, number of children, and climate change anxiety explained 19.5% of the total variance in the sexual quality of life of the participants. In this study, it was determined that the participants had high climate change anxiety levels and low sexual quality of life levels. Climate change anxiety was identified as a predictor affecting the levels of sexual quality of life. Healthcare workers integrate climate-sensitive approaches and behavioral interventions into their sexual health counseling practices.

## 1. Introduction

Climate change and weather events associated with this change affect all aspects of human life.^[1,2]^ The changing climates may result in extreme weather events like heatwaves, storms, droughts, and floods. These events affect the physiological and psychological well-being of people. The physical influence of natural events, especially natural disasters, includes physiological diseases, whereas their psychological effects include conditions such as depression, antisocial behaviors, suicidal ideation, stress, and anxiety.^[3]^ One of the areas in which the bearing of climate change on human well-being and health is the most severe is sexual and reproductive health.^[2]^

Climate change is a significant risk factor for sexual and reproductive health.^[2]^ Climate change is associated with a decrease in the rates of safe sexual behaviors and access to healthcare services.^[3,4]^ The effects of climate change are particularly more severe among low-income and vulnerable populations, as well as women.^[2]^ Women are also more susceptible to the expected consequences of climate change due to the reproductive burden they have and their gender roles.^[5]^

Most people think climate change is a large hazard and feel anxiety regarding its predicted effects. As various disasters such as floods, heatwaves, and wildfires occur due to climate change, the anxieties of people in this context also constantly increase.^[6]^ Moreover, many people have environmental concerns that may affect their sexual health.^[7]^ In the literature, while men were reported to be more optimistic regarding climate change and believe in government policies, women were found to experience more anxiety than men did.^[3,8]^ It was stated that anxieties related to climate change were shaped based on gender differences and the economic development levels of countries, where women in wealthier countries expressed their anxieties more frequently.^[9]^ The effects of the concerns of women, in particular, about this issue on their sexual and reproductive health are highly important in terms of the health of future generations. The number of studies focusing on climate change and sexual and reproductive health together is very limited in the literature. Articles on this topic have focused on the development of a theoretical framework or the effects of natural disasters on access to sexual and reproductive healthcare services.^[2,7–10]^ Additionally, in the literature on climate change and sexual health, researchers have emphasized the lack of sufficient information about this issue and the need for planning studies on the topic.^[4,11]^ In the literature review, no study examining the relationship of climate change anxiety in women with women sexual quality of life could be found. It is believed that this study will fill a significant gap in the literature, and its results will guide future efforts to improve women health. The present study was conducted to identify the relationship between the climate change anxiety levels of women and their sexual quality of life.

## 2. Materials and methods

### 2.1. Design

This study was carried out using a correlational and descriptive research design.

### 2.2. Participants

The study was completed with 952 participants between December 2023 and March 2024. In the post hoc power analysis, which was carried out using in the G*Power 3.1.9.7 (Heinrich-Heine-Universität Düsseldorf, Düsseldorf, Germany) program, for a 95% confidence interval, an effect size of 0.05, 5% margin of error, and a sample of 952 participants, the power of the study was found to be 99%. Women with any health problems, women with a diagnosed psychiatric illness, pregnant women, postpartum women, women with medical/health-related education, and women working at healthcare institutions were excluded.

### 2.3. Data collection

Data were obtained online between December 2023 and March 2024 using the Google Forms platform. Women were sent invitations to participate in the study and the link to the online survey (Facebook/Twitter/Instagram). To ensure that the participants answered all questions, the “required” option was selected for each question on the platform. When a question was left unanswered, the respondent received the following text warning: “this is a required item.” The suitability of the questions on the online survey form was checked by the researchers, who responded to the items themselves before sending them out.

### 2.4. Instruments

The online survey consisted of 3 parts: a personal information form, the Women Climate Change Anxiety Scale, and the Sexual Quality of Life – Female Questionnaire. The Personal Information Form included questions about the sociodemographic and obstetric characteristics of the participants.

Women Climate Change Anxiety Scale (CCAS-W) was created by Söğüt and Avcibay Vurgeç^[12]^ to assess the climate change anxieties of women regarding their health. It is a 5-point Likert-type instrument consisting of 18 items. It has 3 dimensions: physiological health (8 items), behavior (6 items), and gender (4 items). It does not have any inversely scored items. The lowest and highest possible scores on CCAS-W are 18 and 90. Greater scores correspond to greater climate change anxiety levels. Cronbach alpha internal consistency coefficient for the scale was reported as 0.969.^[12]^

Sexual Quality of Life – Female (SQOL-F) Questionnaire was created by Symonds et al to evaluate women sexual quality of life in the last 4 weeks.^[13]^ The reliability and validity of the Turkish form of the scale were tested by Tuğut and Gölbaşi^[14]^. It is a 6-point Likert-type instrument with 18 items. Higher scores show a higher sexual quality of life. Cronbach alpha internal consistency coefficient for the scale was stated to be 0.83.^[14]^

### 2.5. Ethical aspect of the study

Approval to conduct this study was provided by Ethics Committee for Scientific Research in Health Sciences, Necmettin Erbakan University (protocol code 615/ date of approval: 06.12.2023). Before their participation, women read the “electronic informed consent form” presented to them on the 1st screen of the survey form. Those who marked the option “yes” for the statement “I have read and understood the conditions of my participation and consent to participate in the study” at the bottom of the consent form were allowed to respond to the items and questions in the form. To prevent participants from filling out the form multiple times, the “limit to 1 response” option was selected in the settings of the Google Documents platform.

### 2.6. Statistical analysis

The descriptive statistics of the collected data consisted of frequency, percentage, mean, median, minimum, maximum, and standard deviation values. The normal distribution of the values was checked based on whether their skewness and kurtosis coefficients were in the range of ± 1.5. The results showed that the collected data were normally distributed. Pearson correlation tests and stepwise regression analysis were used to test the relationships between variables. For the results of all analyses, *P* < .05 was interpreted as statistically significant. The statistical analyses of the data collected in this study were performed in SPSS V25 (Chicago).

## 3. Results

Most of the participants were 36 years old or older, had university degrees, and were working. The sociodemographic and obstetric features of the participants are given in Table 1. The participants had a mean CCAS-W score of 65.41 ± 11.51 and a mean SQOL-F score of 34.24 ± 14.68 (Table 2). A Pearson correlation analysis was performed on the relationship between the sexual quality of life and climate change anxiety levels of the participants. According to the results of the analysis of their total scale scores, a negative and weak but significant relationship was identified between the sexual quality of life levels and climate change anxiety levels of the participants (*r* = −0.272, *P* < .001). Additionally, the SQOL-F total scores of the participants were significantly and negatively correlated with their CCAS-W physiological health (*r* = −0.265, *P* < .001), behavior (*r* = −0.182, *P* < .001), and gender (*r* = −0.150, *P* < .001) subscale scores. Accordingly, as the climate change anxiety levels of the participants increased, their sexual quality of life levels decreased (Table 3).

**Table 1:** Demographic and obstetric characteristics of the participants (n = 952).

Variables	n	%
Age (yr)		
18–25	179	18.8
26–35	321	33.7
≥36	452	47.5
Education		
Primary-middle school	230	24.2
High school	304	31.9
University	418	43.9
Spouse education		
Primary-middle school	173	18.2
High school	321	33.7
University	458	48.1
Working Status		
Working	513	53.9
Not working	439	46.1
Spouse working status		
Working	905	95.1
Not working	47	4.9
Place of residence		
Village	162	17.0
District	274	28.8
City	516	54.2
Marriage duration		
≤5 yr	374	39.3
>5 yr	578	60.7
Income status		
Income < expenses	153	16.1
Income ~ expenses	446	46.8
Income > expenses	353	37.1
Family type		
Nuclear family	744	78.2
Extended family	208	21.8
Number of pregnancies		
0	190	20.0
1	307	32.2
2 or more	455	47.8
Number of children		
None	217	22.8
1	258	27.1
2 or more	477	50.1
Miscarriage history		
Yes	169	17.8
No	783	82.2
Abortion history		
Yes	66	6.9
No	886	93.1

**Table 2:** CCAS-W and SQOL-F scores of the participants (n = 952).

Scales	Mean ± SD	Min–Max
SQOL-F total	34.24** ± **14.68	18–84
CCAS-W total	65.41** ± **11.51	31–90
CCAS-W subscales		
Physiological health	31.28** ± **6.59	8–40
Behavior	17.84** ± **4.58	2–30
Gender	16.21** ± **3.12	6–20

CCAS-W = Women Climate Change Anxiety Scale, Max = maximum scale score, Min = minimum scale score, SD = standard deviation, SQOL-F = Sexual Quality of Life – Female.

**Table 3:** Correlations between the CCAS-W and SQOL-F scores of the participants.

Variables	1	2	3	4
SQOL-F				
* r*	1			
* P*				
CCAS-W total				
* r*	−0.272***	1		
* P*	<.001			
CCAS-W physiological health				
* r*	−0.265***	0.871***	1	
* P*	<.001	<.001		
CCAS-W behavior				
* r*	−0.182***	0.733***	0.397***	1
* P*	<.001	<.001	<.001	
CCAS-W gender				
* r*	−0.150***	0.730***	0.559***	0.377***
* P*	<.001	<.001	<.001	<.001

CCAS-W = Women’s Climate Change Anxiety, SQOL-F = Sexual Quality of Life–Female.

*** *P*** **<** **.001.

A stepwise regression analysis was carried out to identify the prediction effects of demographic variables and climate change anxiety on sexual quality of life. The 1st step included demographic variables (age, marriage duration, income status, education levels, number of pregnancies, number of children, history of miscarriage, and history of abortion). In the 2nd step, climate change anxiety was introduced to the model. The results of the analysis showed that among the demographic variables included in the model in the 1st step, age (β = 0.146, *P* < .01) and marriage duration (β = 0.191, *P* < .001) were positive significant predictors of sexual quality of life, whereas education level (β = −0.119, *P* < .001) and number of children (β = −0.225, *P* < .001) were significant and negative predictors of sexual quality of life. Income status, abortion history, and miscarriage history did not have any significant effect on the sexual quality of life levels in the sample (*P* > .05). It was determined that climate change anxiety, which was included in the model in the 2nd step after controlling for demographic variables, significantly and negatively predicted sexual quality of life (β = −0.234, *P* < .001). Age, marriage duration, education status, number of children, and climate change anxiety collectively explained 19.5% of the total variance in the sexual quality of life levels of the sample (Table 4).

**Table 4:** Predictive effects of demographic variables and climate change anxiety on sexual quality of life.

Variables	*R*	Adjusted *R*^2^	*B*	SE	*F*	*P*	β	*t*	*P*
(Constant)	0.450	0.195	49.143	5.750	26.585	<.000		8.546	**<.001**
Age			0.243	0.079			0.148	3.061	**.002**
Marriage duration			0.280	0.089			0.170	3.145	**.002**
Education			−2.294	0.550			−0.129	−4.167	**<.001**
Income status			−0.866	0.631			−0.041	−1.374	.170
Number of pregnancies			2.861	1.040			0.200	2.752	**.006**
Number of children			−3.079	0.988			−0.215	−3.118	**.002**
Miscarriage history			1.203	1.302			0.031	0.924	.356
Abortion history			0.353	1.894			0.006	0.186	.852
CCAS-W			−0.298	0.038			−0.234	−7.910	**<.001**

The significance of the bold values has been clarified. Bold values represent statistically significant predictors (*P* < .05).

β = standardized beta, *B* = non-standardized beta, CAS-W = Women’s Climate Change Anxiety Scale, *P* = significance, SE = standard error, *t* = significance of beta.

## 4. Discussion

This study was conducted to identify the relationship between the climate change anxiety levels and sexual quality of life in women. The results showed that the participants had high of climate change anxiety levels, and this situation adversely affected their levels of sexual quality of life. Climate change anxiety was identified as a predictor affecting the participants in terms of their sexual quality of life levels. In the literature, varying levels of climate change anxiety have been observed among women.^[15,16]^ Consistent with the present findings, studies conducted in China, India, Japan, and the United States,^[17]^ in the USA,^[18]^ in African and European countries,^[15]^ have also documented heightened levels of concern about climate change among women. Demir et al found that women displayed moderate degrees of anxiety regarding climate change.^[19]^ Among studies carried out with adults, women were determined to have very low climate change anxiety levels in Germany,^[20]^ United Kingdom,^[21]^ and Poland.^[22]^The majority of the participants of this study had high levels of education (33.7%: high school, 48.1%: university). As the education levels of individuals increase, their awareness/knowledge regarding climate change may also increase. This situation may have raised the anxieties of the participants related to climate change Therefore, it is highly important to organize climate change education programs targeting women well-being and to empower women with the necessary knowledge, skills, values, and attitudes in this regard, regardless of their educational background.^[23]^ Furthermore, changes in living conditions are a source of stress for all living organisms.^[1]^ The effects of climate change such as increased temperatures and disasters are at highly noticeable levels, and they may affect individuals in different ways. This may lead to stress in these individuals. The anxiety levels of the participants of this study may have risen as a response to this stress.

The sexual quality of life levels of the participants in this study was found to be low. In contrast, women quality of life related to sexuality has been reported to be moderate in some studies^[24,25]^ and above moderate in others.^[26,27]^ Some studies have shown “good” levels of sexual quality of life among women.^[28,29]^ This difference between the findings obtained in this study and those reported in others in the literature may be explained by differences in measurement instruments and the sociodemographic characteristics of samples.

The age of the participants, their marriage duration, education status, number of children, and climate change anxiety levels explained 19.5% of the total variance in their SQOL-F scores. Çataloluk and Eroğlu reported that women educational status and employment status, the education and employment statuses of their husbands, their perceived income levels, type of marriage, mode of delivery, and self-perceptions of weight explained 25% of the total variance in their SQOL-F scores.^[30]^ Panahi et al observed that sexual quality of life in women was affected by their age of marriage, education levels, weekly frequency of sexual intercourse, and sexual health literacy.^[31]^ Barikani et al reported a significant association between marriage duration and sexual functioning, and a 1-year increase in marriage duration corresponded to a 0.25-unit decrease in sexual functioning.^[32]^ The differences in the results of this study and those reported in others in the literature may be attributed to differences in measurement instruments and samples.

The variables that influence sexual quality of life are highly diverse.^[33,34]^ In this study, climate change anxiety was found to be one of these factors. According to Logie et al, climate change and sexual health were significantly associated, and this situation could increase social inequalities.^[4]^ It was reported that the exposure of individuals to extreme weather events, vector-borne disease risks, and other effects of climate change will increase over time.^[35]^ For this reason, although the findings of this study showed a weak relationship between climate change anxiety and sexual quality of life, 1 may expect that this relationship will become stronger as the effects of climate change increase over time.

In addition to the quantitative evidence presented in this study, future research could adopt a mixed-methods approach. In particular, qualitative interviews may help to elucidate individuals’ personal experiences and contextual factors that underlie the observed associations. Such insights could capture nuances that are often overlooked in survey-based studies, including cultural beliefs, gender roles, and community-level dynamics.^[36–38]^

The findings also carry important implications for public health policy. For instance, the evidence highlights the need for gender-sensitive climate education and awareness programs that explicitly address women well-being. Incorporating these results into health promotion strategies may contribute to more inclusive interventions, particularly in settings where women face disproportionate vulnerabilities to climate-related stressors.^[39–41]^

Finally, situating these findings within a global context is essential. Similar studies conducted in South Asia and Sub-Saharan Africa have reported comparable gendered patterns, whereas research from Western Europe has emphasized different cultural determinants. Comparing the present results with international evidence highlights the variability of climate-health perceptions across cultural contexts, underscoring the importance of tailoring interventions to local needs.^[42–44]^

### 4.1. Limitations

This study had some limitations. First of all, the data of the study were collected online. This may have led to the exclusion of women with lower e-literacy or no access to the internet. On the other hand, considering the highly private state of sex-related topics in Turkish society and the sensitive nature of the topic, it was thought that conducting the study online would prevent response bias. Second of all, the results may only be generalized to women in the age group of 20 to 49. A limitation of the study was the highly limited number of studies covering both climate change and sexual and reproductive health.^[2,10]^ Articles on these topics have mainly focused on the development of a theoretical framework or the effects of natural disasters on access to sexual and reproductive healthcare services.^[2,10]^ Thus, the comparability of the findings of this study was limited. Nevertheless, this study can highlight the necessity to conduct more studies on the topic, which was another strong aspect of the study.

## 5. Conclusion

The data obtained and analyzed in this study offered new information about the relationship between women climate change anxiety levels and sexual quality of life. It is believed that these results are important in terms of the integration of climate-sensitive decisions and behavioral interventions into sexual health counseling practices by healthcare workers. In this context, healthcare workers may be recommended to plan education programs for women about the effects of climate change on sexual and reproductive health. These education programs may make it possible to improve women health by raising awareness regarding the damaging impacts of climate change on women health. Future studies can utilize mixed-method designs by including in-depth interviews. More detailed information about climate change anxiety and sexual quality of life can be obtained this way.

## Acknowledgments

The authors thank to women who participated to this study.

## Author contributions

**Conceptualization:** Hamide Aygör, Necibe Şeyda Tunali, Yusuf Bayar.

**Data curation:** Hamide Aygör, Necibe Şeyda Tunali.

**Formal analysis:** Yusuf Bayar.

**Investigation:** Hamide Aygör.

**Methodology:** Hamide Aygör, Necibe Şeyda Tunali, Yusuf Bayar.

**Project administration:** Hamide Aygör.

**Supervision:** Hamide Aygör.

**Writing – original draft:** Hamide Aygör, Necibe Şeyda Tunali, Yusuf Bayar.

**Writing – review & editing:** Hamide Aygör, Necibe Şeyda Tunali, Yusuf Bayar.

## References

[1] Sofuoglu Z Cankardaş S. Review of climate change and its effects on the individual. J Nesne Psychol. 2021;9:139–46.

[2] Segal TR Giudice LC. Systematic review of climate change effects on reproductive health. Fertil Steril. 2022;118:215–23. 10.1016/j.fertnstert.2022.06.005 35878942

[3] Cianconi P Betrò S Janiri L. The impact of climate change on mental health: a systematic descriptive review. Front Psychiatry. 2020;11:74. 10.3389/fpsyt.2020.00074 PMC7068211 32210846

[4] Logie CH Toccalino D Reed AC. Exploring linkages between climate change and sexual health: a scoping review protocol. BMJ Open. 2021;11:e054720. 10.1136/bmjopen-2021-054720 PMC8524293 34663670

[5] Sorensen C Murray V Lemery J Balbus J. Climate change and women’s health: impacts and policy directions. PLoS Med. 2018;15:e1002603. 10.1371/journal.pmed.1002603 PMC6038986 29990343

[6] Hickman C Marks E Pihkala P. Climate anxiety in children and young people and their beliefs about government responses to climate change: a global survey. Lancet Planet Health. 2021;5:e863–73. 10.1016/S2542-5196(21)00278-3 34895496

[7] Rousseau C. Climate change and sexual and reproductive health: what implications for future research? Sex Reprod Health Matters. 2023;31:2232196. 10.1080/26410397.2023.2232196 PMC10444000 37594319

[8] Shoko Kori D. The psychosocial impact of climate change among smallholder farmers: a potential threat to sustainable development. Front Psychol. 2023;14:1067879. 10.3389/fpsyg.2023.1067879 PMC10169624 37179880

[9] Bush SS Clayton A. Facing change: gender and climate change attitudes worldwide. Amer Polit Sci Rev. 2023;117:591–608.

[10] Ashraf M Shahzad S Sequeria P Bashir A Azmat SK. Understanding challenges women face in flood-affected areas to access sexual and reproductive health services: a rapid assessment from a disaster-torn Pakistan. Biomed Res Int. 2024;2024:1–13. 10.1155/2024/1113634 PMC11001467 38590384

[11] Stephenson R Gonsalves L Askew I Say L. Detangling and detailing sexual health in the SDG era. Lancet (London, England). 2017;390:1014–5. 10.1016/S0140-6736(17)32294-8 28877852

[12] Söğüt D Vurgeç BA Atik AD. Development of the climate change anxiety scale for women’s health. In: International Congress of Climate Change Effects on Health Life, Engineering and Social Sciences Proceeding Book. Konya, Turkey; 2022. Abstract 173.

[13] Symonds T Boolell M Quirk F. Development of questionnaire on sexual quality of life in women. J Sex Marital Ther. 2005;31:385–97. 10.1080/00926230591006502 16169822

[14] Tuğut N Gölbaşi ZA. Validity and reliability study of Turkish version of the sexual quality of life questionnaire-female. Cumhuriyet Med J. 2010;32:172–80.

[15] Heeren A Daouda CM McNally RJA. Network approach to climate change anxiety and its key related features. J Anxiety Disord. 2023;93:102625. 10.1016/j.janxdis.2022.102625 36030121

[16] Regnoli GM Tiano G De Rosa B. Is climate change worry fostering young Italian adults’ psychological distress? an Italian exploratory study on the mediation role of intolerance of uncertainty and future anxiety. Climate. 2024;12:118. 10.3390/ejihpe14060121 PMC11202537 38921087

[17] Tam KP Chan HW Clayton S. Climate change anxiety in China, India, Japan, and the United States. J Environm Psychol. 2023;87:101991.

[18] Clayton SD Pihkala P Wray B Marks E. Psychological and emotional responses to climate change among young people worldwide: differences associated with gender, age, and country. Sustainability. 2023;15:3540.

[19] Demir R Yalazi RO Dinç A. The relationship between women’s climate change awareness and concerns about climate change in Turkey. Public Health Nurs. 2024;41:215–20. 10.1111/phn.13269 38041428

[20] Hajek A König HH. Climate anxiety in Germany. Public Health. 2022;212:89–94. 10.1016/j.puhe.2022.09.007 36272204

[21] Whitmarsh L Player L Jiongco A. Climate anxiety: what predicts it and how is it related to climate action. J Environm Psychol. 2022;83:101866.

[22] Larionow P Sołtys M Izdebski P. Climate change anxiety assessment: the psychometric properties of the polish version of the climate anxiety scale. Front Psychol. 2022;13:870392. 10.3389/fpsyg.2022.870392 PMC9130850 35645848

[23] United Nations Educational, Scientific and Cultural Organization. Climate Change Education. 2023. https://www.unesco.org/en/climate-change/education. Accessed January 2, 2025.

[24] Panahi R Anbari M Javanmardi E. The effect of women’s sexual functioning on quality of their sexual life. J Prev Med Hyg. 2021;62:776–81. 10.15167/2421-4248/jpmh2021.62.3.1945 PMC8639126 34909508

[25] Yaşar Ayan M Beydağ KD. Determination of the relationship between genital self-image and sexual quality of life in sexually active women. Cauc J Sci. 2023;10:115–24.

[26] Kaplan SE Duman M Yilmaz S. Sexual life quality and marital adjustment in women with and without diabetes. Sex Disabil. 2020;38:625–35.

[27] Akalin A Bostanci S. Sexual functions and sexual quality of life in the reproductive age women using method of family planning. Androl Bul. 2022;24:110–7.

[28] de Hollanda GSE Nogueira WP de Lima Barroso BI. Quality of sexual life of riparian women: analysis of sexual practices and attitudes. Enferm Clin. 2022;32:405–12. 10.1016/j.enfcle.2022.04.006 35598872

[29] Khorshidi M Alimoradi Z Bahrami N Griffiths MD. Predictors of women’s sexual quality of life during the COVID-19 pandemic: an Iranian cross-sectional study. Sexual Relationship Ther. 2022;39:444–57.

[30] Çataloluk A Eroğlu V. The relationship between genital self-image and the quality of sexual life in married Turkish women. Sexuality Culture. 2025;29:191–209.

[31] Panahi R Kheiri M Daronkolaei ZA. The effect of sexual health literacy on the sexual life quality of women referring to healthcare centers in Qazvin, Iran. J Educ Health Promotion. 2021a;10:391. 10.4103/jehp.jehp_1484_20 PMC8641725 34912927

[32] Barikani A Kia MS Khoshkchali A. Relationship between health literacy level and sexual function in women in the northwest of Iran in 2020 – a cross-sectional study. BMC Womens Health. 2023;23:176. 10.1186/s12905-023-02322-2 PMC10091605 37041642

[33] Bagherinia M Dolatian M Mahmoodi Z Ozgoli G Alavi Majd H. Evaluating the relationship between structural determinants of health and quality of sexual life in women: a systematic review. Crescent J Med Biol Sci. 2022;9:184–8.

[34] Petruccelli F Diotaiuti P Verrastro V. Affective dependence and aggression: an exploratory study. Biomed Res Int. 2014;2014:1–11. 10.1155/2014/805469 PMC4094873 25054147

[35] Mendola P Ha S. Beyond the infant in your arms: effects of climate change last for generations. Fertil Steril. 2022;118:224–9. 10.1016/j.fertnstert.2022.06.007 35791979

[36] Creswell JW Clark VLP. Designing and Conducting Mixed Methods Research. 3rd ed. Sage Publications; 2017.

[37] Fetters MD Curry LA Creswell JW. Achieving integration in mixed methods designs – principles and practices. Health Services Res. 2013;48:2134–56. 10.1111/1475-6773.12117 PMC4097839 24279835

[38] O’Cathain A Murphy E Nicholl J. Three techniques for integrating data in mixed methods studies. BMJ. 2010;341:c4587. 10.1136/bmj.c4587 20851841

[39] Clayton S Manning C Krygsman K. Mental Health and Our Changing Climate: Impacts, Implications, and Guidance. American Psychological Association & EcoAmerica; 2017.

[40] World Health Organization. Gender, climate change and health. WHO. 2021. https://www.who.int/publications/i/item/9789240038363. Accessed January 2, 2025.

[41] van Daalen KR Jung L Dhatt R Phelan AL. Climate change and gender-based health disparities. Lancet Planetary Health. 2020;4:e44–5. 10.1016/S2542-5196(20)30001-2 32112742

[42] Haynes K Tanner TM. Empowering young people and strengthening resilience: youth-centred participatory video as a tool for climate change adaptation and disaster risk reduction. Children’s Geographies. 2015;13:357–71.

[43] Tschakert P Machado M. Gender justice and rights in climate change adaptation: opportunities and pitfalls. Ethics Soc Welfare. 2012;6:275–89.

[44] van Daalen KR Oron AP El Omrani O. Climate change and gender-based health disparities. Lancet Planetary Health. 2022;6:93–5. 10.1016/S2542-5196(20)30001-2 32112742

